# Echo chamber effects on short video platforms

**DOI:** 10.1038/s41598-023-33370-1

**Published:** 2023-04-18

**Authors:** Yichang Gao, Fengming Liu, Lei Gao

**Affiliations:** 1 Business School, Shandong Normal University, Ji’nan, 250014 China; 2 Commonwealth Scientific and Industrial Research Organisation (CSIRO), Waite Campus, Urrbrae, SA 5064 Australia

**Keywords:** Scientific data, Information technology

## Abstract

In recent years, short videos have become an increasingly vital source of information. To compete for users’ attention, short video platforms have been overusing algorithmic technology, making the group polarization intensify, which is likely to push users into the homogeneous “echo chamber”. However, echo chambers can contribute to the spread of misleading information, false news, or rumors, which have negative social impacts. Therefore, it is necessary to explore echo chamber effects in short video platforms. Moreover, the communication paradigms between users and feed algorithms greatly vary across short video platforms. This paper investigated echo chamber effects of three popular short video platforms (Douyin, TikTok, and Bilibili) using social network analysis and explored how user features influenced the generation of echo chambers. We quantified echo chamber effects through two primary ingredients: selective exposure and homophily, in both platform and topic dimensions. Our analyses indicate that the gathering of users into homogeneous groups dominates online interactions on Douyin and Bilibili. We performed performance comparison of echo chamber effects and found that echo chamber members tend to display themselves to attract the attention of their peers and that cultural differences can prevent the development of echo chambers. Our findings are of great value in designing targeted management strategies to prevent the spread of misleading information, false news, or rumors.

## Introduction

Ranging from a few seconds to a few minutes, short videos have become a popular form of human’s entertainment and content sharing. Influenced by the attention economy, short video platforms have started to abuse feed algorithms to compete for users’ attention. Feed algorithms classify users’ preferences by collecting their behavioral data, thus matching users with precise and continuous information. This matching of information gradually creates a powerful driving force for group polarization, which is highly likely to lead to the formation of echo chambers. In addition, due to the nature of such short video platform that spreads information quickly and widely^1,2^, there have been attempts to disseminate misleading information^3^, false news^4^, or rumors^5^. According to recent studies, the echo chamber effect of social media can promote the spread of misleading information, fake news, and rumors^6–8^. Such information usually harms people, society, or economics.

Echo chamber (EC) is one of the central areas of research in the digital age, spanning several disciplines including psychology, political science, and information science^9^. Social media has three main features that make it the perfect environment for ECs: no geographical restrictions, no social cost to sharing fringe beliefs, and fellow believers can be found no matter how fringe^10^. Previous research on online ECs has focused on political fragmentation and polarization in democracies^11,12^, the impact on information dissemination^7,13–15^, and EC identification models^16–18^. As a new type of social media, because of the scarce research on user behavior features and news consumption of short video platforms, the effects and the very existence of ECs have been questioned. In this situation, the debate around ECs is critical to understanding the impact of short video platforms on news consumption and public opinion formation.

In this paper, we explored key differences between short video platforms as well as user features, and how they may or may not influence the formation of ECs. As recently shown in the research on short videos, multiple platform studies can provide a new perspective on long-debated issues. We have selected three newly popular short video platforms for our study: Douyin, TikTok and Bilibili, and designed a comparison of topic dimensions. We introduced an operational definition of an EC and applied social network analysis to provide a common methodological basis to explore how different platforms influence their formation. In particular, we measured the two common elements that characterize ECs into observables that can be quantified and empirically measured: selective exposure and homophily. Then, we used these elements to compare the EC effect in terms of platform dimension and topic dimension in the following four aspects: EC size, EC type, selective exposure, and homophily. Our findings indicated that users clustered in homogeneous groups dominated online interactions on Douyin and Bilibili and that there is greater racial segregation on Douyin. Furthermore, the pattern of information dissemination within the EC is similar over time, independent of differences in platforms and topics. From the analysis of short video EC members who share common views or interests, reveals that they prefer to showcase themselves on the platform and hence elicit others’ participation. Furthermore, by analyzing user interaction data from various nations, we discovered that cultural differences among platform users could prevent the development of ECs.

The rest of this paper is organized as follows. “Data and method” section describes in detail the source of the data set, the operational definition of the EC and the EC effect metric. “Results” section shows the comparison results of the EC effect in different dimensions and the feature analysis associated with EC members. “Discussions” section discusses the research results. Finally, “Conclusion” section concludes our research work and summarizes the strengths, limitations, and possible future research directions of this work.

## Data and method

### Framework of the methodology

**Figure 1:**
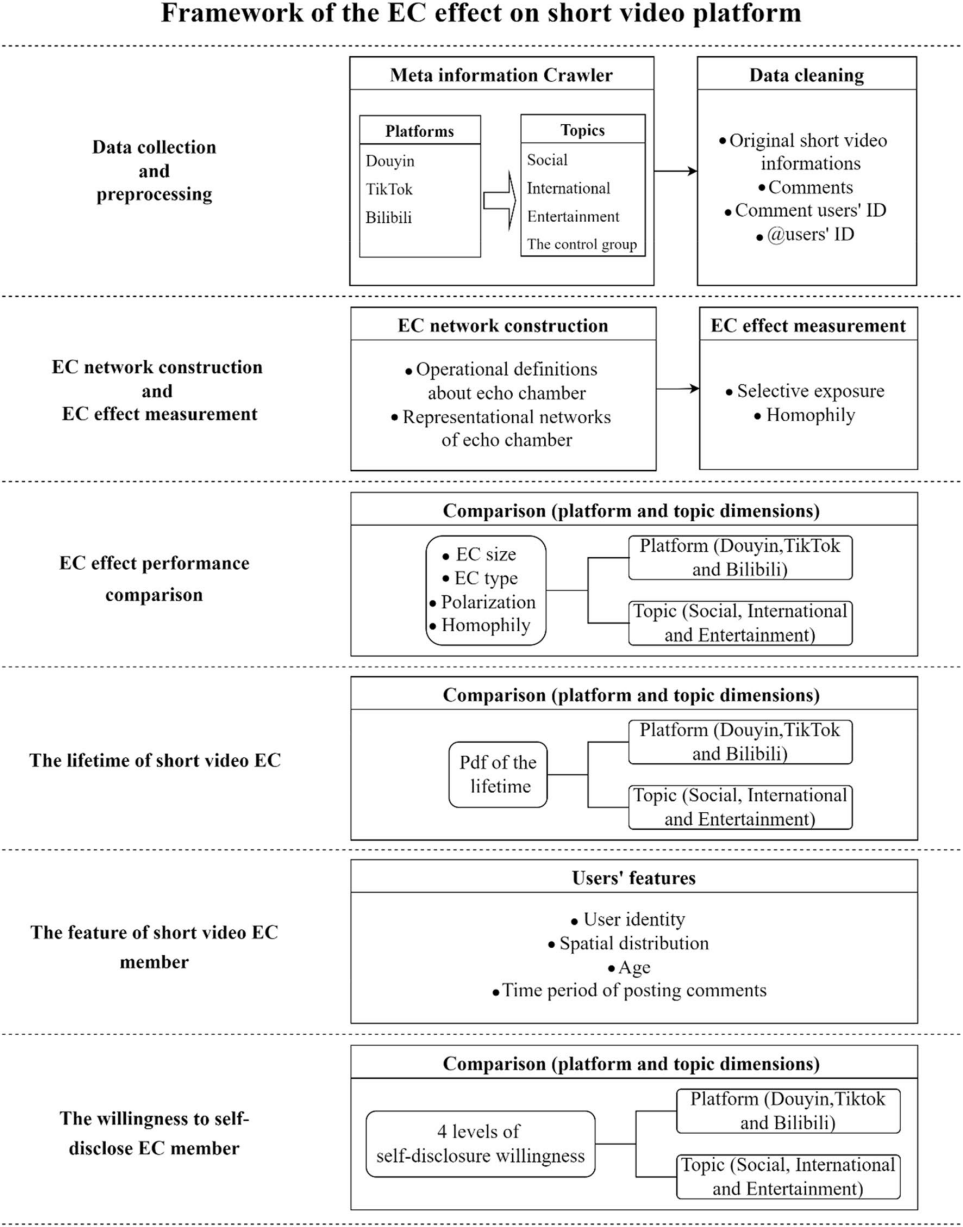
Framework of the methodology. The methodology in this work consists of six steps: (1) data collection and preprocessing, (2) EC network construction and EC effect measurement, (3) EC effect performance comparison, (4) the lifetime of short video EC, (5) the feature of short video EC member, and (6) the willingness to self-disclose of short video EC member.

The framework of the methodology is shown in Fig. 1. The methodology starts with the collection and analysis of short video information. Based on the collected raw data, the original short video and the comment text, along with its commenting and @ users’ IDs, are retained through data cleaning. *In the second step*, we give the operational definition of the EC. The EC is defined as a group of users who are involved in commenting on at least two short videos on the same topic, while including users who were @ by EC members in the same EC. To explore the key differences between the different ECs, we divide them into three types according to their main sentiment tendencies: positive, negative and controversial, respectively. We then measure the presence of an EC effect on short video platforms in terms of two critical features known to ECs. *In the third step*, we compare the EC effect in terms of platform dimension and topic dimension in the following four aspects: EC size, EC type, selective exposure and homophily, respectively. *In the fourth step*, we investigate the impact of ECs on short video propagation. So we compare the lifetime of ECs across platforms and topics. *In the fifth step*, we measure the difference between EC members and non-members from four perspectives: user identity, spatial distribution, age, and commenting time. *Finally*, we explore the relationship between users’ willingness to self-disclose and EC members. The detailed methodology and results are described in “Data collection and preprocessing”-“The willingness to self-disclose of short video EC member” sections.

### Data collection and preprocessing

“Short video” refers to video material that is less than 5 min long and is disseminated through digital media outlets^19^. The boundary between producers and consumers of short videos is blurred, the production cost is low, and the content is easy to consume^20^. Short video platforms have steadily evolved from primarily entertainment platforms to new platforms with social network features that live and grow with users as they continue to better themselves^19^. The proliferation of short video-sharing mobile apps has led to new forms of information-sharing practices. Even the traditional social media platforms have been influenced by these popular video-sharing mobile apps because some people are drawn away from watching the news to watching short videos^21–23^.

Given the diversity of online customers, we selected the following short video platforms for our study:

Douyin (https://www.Douyin.com/), a short video app for musical creativity incubated by ByteDance^20,24^. The app was launched in 2016, is a short video community platform for all ages. Users can share their lives through the platform, but also here to meet more friends, to understand a variety of strange and interesting things^25–27^.

TikTok (https://www.TikTok.com/) is the worldwide counterpart to the Chinese mobile short video app Douyin. The digital structure of TikTok is similar to that of Douyin, but it serves to an entirely different group of users and is governed by distinct forces. TikTok’s regionalization approach varies from that of previous social media platforms in that its user base is global in nature, and its objective is to make its product adhere to the various expectations, customs, and legislative frameworks of several nations^20,28^.

Bilibili (https://www.bilibili.com/), a Chinese top youth culture community and video platform, is an early video site for creating and sharing of ACG (animation, comics, games) material^29^. After more than a decade of development, the site has developed a big multicultural community around its users, artists, and content^30^. Technically speaking, Bilibili cannot be considered as a short video platform. However, it has a short video section and is popular with the majority of internet users^30–32^.

To identify and analyze ECs in short video platforms, we established the data collection system. The resulting dataset is divided into two parts: experimental and control groups. The experimental group contains three common topics: social, international, and entertainment. The control group is a randomly selected pan-topic dataset in each platform. To ensure the validity of the control group, the control group dataset is of the same order of magnitude as the experimental group. The selection of topic events is based on the event library section of the “Zhiweidata” platform (https://ef.zhiweidata.com/library). As shown in Table 1, we search for all social, international and entertainment events from July 1, 2022 to July 30, 2022, and rank them according to the influence index. Finally, we selected the top 1 events under each topic as our event set. “Zhiweidata” is a famous comprehensive data analysis platform of hot events on the internet in China, with in-depth and objective interpretation of the truth of the events^33^. This website has accumulated a vast amount of hot events, involving ten categories: international, social, corporate, internet, financial, entertainment, sports, government, disaster, and crime.

**Table 1:** Top 5 of events in the Zhiweidata platform under three topics.

No	Topics					
	Social		International		Entertainment	
	Events	Index	Events	Index	Events	Index
1	Hong Kong launches activities to celebrate the 25th anniversary of Hong Kong's return to China	83.1	Pelosi’s visit to Taiwan	89.8	Jay Chou’s new album "The Greatest Hits" first song released	78.6
2	Successful launch of the QTS experiment module	81.1	Former Japanese Prime Minister Shinzo Abe shot and killed	86.7	Yi Yang Qianxi Luo Yi Zhou Hu Xianxu got into the National Theatre	78.1
3	Jiangxi a state-owned enterprise employees in the circle of friends show off rich show right	75.1	British Prime Minister Boris Johnson agrees to resign	75.3	Taiwan media exposed Lin Zhiying father and son in a car accident	77.8
4	A 22-year-old girl in Hangzhou died suddenly after continuous overtime work	72.9	Positive biden new crown test	70.6	Jia Nai Liang apologizes for controversial cooperation with Funky	68.7
5	Multiple people diagnosed with pyrexia in multiple locations	72.2	Sri Lanka's president officially announced his resignation	66.7	MIRROR concert stage accident	67.9

We developed three Python crawlers to gather the raw information of the platforms, including the list of comments and user information of comments/@. To eliminate the possible influence of time period, we limited the time of data collection to 24:00 from the beginning of the event to the 30th day. Furthermore, we randomly collected the same number of pan-topic informations during the same time period on each platform as control groups. And the control groups were required to have approximately the same number of commenting/@ users to exclude the effect of the number of users involved in the interaction on the study.

Within the collected information, there were often meaningless special symbols, hyperlinks, and distracting information posted by paid internet trolls and bot accounts, which we must clean up to obtain a standardized text dataset. Data cleaning is separated into three steps: (1) reduce data noise via data filtering, removing duplicates, deleting blank and invalid text, removing hyperlinks, (2) eliminate meaningless ultrashort tweets and tweets with few retweets, and (3) use the Socialbothunter algorithm^34^ to identify and remove distracting information posted by paid internet trolls and bot accounts^35^.

### EC network construction

The EC effect was first identified in research in politics and is now widely validated in the study of social networks^35–37^. An echo chamber is a group in which users’ opinions, political leanings, or beliefs about a topic are reinforced by repeated interactions with peers with similar tendencies and attitudes, and they tend to gravitate toward information that supports their beliefs and join in around a common discussion^36,38,39^. On social networks, influenced by selective exposure and confirmation bias, people tend to join clusters that conform to their beliefs, leading to the creation of homogeneous and polarized communities “ECs”. The concern over the potentially harmful effects of online social media on society sparked study interest in online ECs. In recent years, research on the spread of fake news and rumors, as well as the global Covid-19 pandemic, has pushed the EC into a new wave of research.

Research on the causes of ECs has suggested that, in addition to confirmation bias, cognitive dissonance related to human psychology, and homophily, recommender algorithms may have played an essential role in the development of ECs^40^. Users are ensnared in tailored information by recommender algorithms, which utilise users’ prior activity to customise recommendations to their preferences^10^. A distinct information space is therefore created around each of us as a result of the ongoing creation and improvement of hypotheses about who you are and what you will do next by these predictive algorithms. Short video platforms have gradually increased their respective user stickiness by utilizing unique recommender algorithms based on brand positioning, such as Douyin and TikTok’s Today’s Headlines algorithm based on content interaction and Bilibili’s recommender algorithm based on community interest. Therefore, it is a fascinating study to explore whether the EC effect exists in short video platforms as well as in traditional social media.

#### Operational definitions about EC

To investigate the key differences between short video platforms, it is necessary to establish a formal definition of an EC. In general, an EC is a closed system or a group of users who share common interests and actively share information with others, resulting in assimilation or even amplification of opinions or memes. First, we need to identify the leaning of users at a micro level. The existing research viewed an EC as a community of people with similar viewpoints. However, determining the orientation of an individual user can be challenging, given that it is considered privacy-sensitive information. In this paper, we define an EC as a group of users who have participated in commenting at least two short videos on the same topic^7^. Given a network describing users’ interactions centered on a common topic, an EC is a subset of the network users who share the same point of view and tend to have dense connections primarily within the same group^7^. An EC network can be defined by a graph $$ G = \left( {V, \, E} \right)$$, where $$V = \left\{ {v_{1} , \ldots ,v_{n} } \right\}$$ is the set of ECs and $$ e(v_{i} ,v_{j} ) \in E$$ is a set of users’ edges which participate in both of the ECs ($$v_{i}$$ and $$v_{j}$$). Since a pair of ECs can share a common event, we only consider the edges where a pair of ECs are identified from disjoint events. In this paper, we considered EC networks with undirected, unweighted edges, i.e., $$e\left( {v_{i} ,v_{j} } \right) \ge 1$$ if $$v_{i}$$ and $$v_{j}$$ have a common event (0 otherwise), and $$ e(v_{i} ,v_{j} ) = e(v_{j} ,v_{i} )$$, respectively^41^. It is important to note that a user can participate in multiple ECs, each with at least two members. Since different forms of communication on social media tend to have different effects. The retweet function plays a crucial role in promoting polarization, and comments make an important contribution in building consensus^42^. However, the @ function is often used to initiate a conversation with a targeted user^43^. Therefore, we included the @user mentioned by the echo chamber members in the same EC.

The ideal state of community communication is to be at a point of equilibrium. A strong polarization occurs when the similarity or difference reaches a certain threshold. Then the EC effect is highly likely to occur in this case. Community polarization can lead to conflict, but conflict is not always negative^44^. Sentiments have been discovered to influence the focus of users’ information searches as well as the information sources they select^45^. While negative sentiments prevent further information searching, positive sentiments cause positive information-seeking actions^46,47^.

To explore the key differences between different ECs, we first identify the ECs using the EC-model algorithm^6^. Then, the ECs are classified into three types according to their dominant affective tendencies. We use the content sentiment polarity of EC members as the judgment criterion: EC members with more than 50% positive sentiment are judged as Positive, with more than 50% negative sentiment are judged as Negative, and others are judged as Controversial ECs. The method of sentiment analysis is detailed in “Selective exposure” section.

### EC effect measurement

In social science literature studies, ECs have the following important characteristics: First, users are selectively exposed to mostly attitudinally consistent content whose expression reinforces users’ pre-existing perceptions. Second, users are surrounded by homogeneous networks whose members are relatively similar in their attitudes or other features^48^. Both selective exposure and homophily can be endogenous, i.e., the result of users’ own active choices, but to some extent they can also be exogenous, i.e., they can be selectively recommended to users by algorithms. In this paper, we will verify whether EC effects of these natures are also present in short video platforms from these two aspects.

#### Selective exposure

Selective exposure, which is thought to be a distinctive feature of (political) echo chambers, is a social phenomenon where a user tends to consume information only that he or she would like to believe. Some claimed that selective exposure dominates content consumption on social media and that different social media platforms may trigger very different dynamics^49^. The literature on selective exposure emphasizes that social networks offer more conflicting perspectives than interpersonal conversations. Social media users tend to consume media content that aligns their preferences if given the option to do so^50^. In view of this, we devised a method to determine whether there is selective exposure in an EC. Specifically, the degree of selective exposure of users is measured by analyzing the sentiment and users’ stance polarity of the comment texts of EC members^51–53^.

Sentiment analysis is a technology based on natural language processing (NLP) and text mining to classify text content with individual subjective sentimental tendencies of users. Sentiment analysis work related to online comments is generally divided into chapter-level, sentence-level, and word-level, depending on the scale of the research object. Chapter-level is suitable for expressing overall sentiment tendencies. Sentence-level assumes that each sentence expresses a single opinion tendency for only one entity and is not applicable to multi-feature documents. Word-level does not focus on text or sentences, but mainly investigates how to locate and analyze the sentiment of feature attributes^54–56^. Short video platforms generally limit comments to 100 characters or less (Douyin 100 characters, TikTok 150 characters, Bilibili 30 characters). The length of a user’s online comment may be a word or a paragraph, depending on individual preferences. Thus, it was challenging to select the best way to analyze the sentiment of online user comments accurately.

In prior research, different kinds of NLP methods have been used for sentiment analysis of text data. Dictionary-based approaches represented by the LIWC and Hedonometer projects match the words that make up each text entry to a specific list of sentiments, which is widely used in social media research. Recently, neural network-based sentiment classification of text data like transformers or convolutional neural networks (CNN) has been successfully implemented. Moreover, they have overcome the limitation of text length and shown good performance^57,58^. In our study, we used a pre-trained multilingual BERT model, Sentence-BERT^59^. The pre-trained model of BERT has been effectively used in multiple areas. The input size is decreased by the model’s independent encoding and saving of entity records. Unlike conventional techniques, BERT generates dynamic word representations that are locally influenced by nearby situations^60^.

We use the trained Sentence-BERT to rate the user comment text in the EC. And we set the output to take values in the range [− 1, 1], where − 1 represents absolute negative sentiment, 1 represents absolute positive sentiment, and 0 represents neutral. However, in our actual study, we find that the majority of @users did not post comments, so we can not classify their sentiment polarity. In view of this, we refer to the average sentiment polarity score of @users’ 10 most recent comments on the same topic as the polarity of this user. Others are marked as unknown.

Stance detection is a key component of many tasks in the research field of social media, e.g., fake news detection^61^, argument mining^62^ and identifying users with opposing views in debates^63^. Stance detection, also known as stance classification, is a popular study topic in Natural Language Processing (NLP). Most studies use stance detection at the statement level to predict a piece of text’s attitude towards a certain problem. In this paper, we utilized a multi-task stance detection model, which effectively integrates syntactic features of sentences into the model^64^. We set the output to take values in the range [− 1, 1] (absolute disagree to absolute agree). And we refer to the average score of @users’ 10 most recent comments on the same topic as the stand of this user. Others are marked as unknown.

In the interactive network, we represented a piece of user-generated content as a sentiment/stance tendency, i.e., the user-generated sentiment/stance tendency $C_{k}$ was a discrete value, taking values between [− 1, 1]. Consider a user $i$ who created $k$ different pieces of content, $C_{i} = \left\{ {C_{1} ,C_{2} , \ldots ,C_{k} } \right\}$, where $k$ was the activity of user $i$, and each sentiment/stance polarity was assigned a numeric value. Then the individual sentiment/stance polarity of user $i$ can be defined as the average of the polarities of produced contents^65^,

1 $$ x_{i} \equiv \frac{{\mathop \sum \nolimits_{j = 1}^{k} c_{j} }}{k} $$

#### Homophily

Homophily is defined as the tendency of individuals to associate with people who share their views or have similar orientations^66^. We devise a method to measure heterogeneity in this work to investigate if homophily occurs among the participants in the EC. It is defined as the balance between members who hold pro- and anti-opinion views^66^. The measurement method is shown in Eq. (2):

2 $$ H = 1 - \frac{{\left| {S - O} \right|}}{{\left| {S + O} \right|}} $$

where $S$ is the observed frequency of positive/agree sentiment/stance among EC members, and $O$ is the observed frequency of negative/disagree sentiment/stance. The metric returns a linear range from 0 (i.e., perfect homogeneity, with only supporters or detractors) to 1 (i.e., perfect heterogeneity, with equal proportions of supporters and detractors). We finally calculated the average of both scores (sentiment and stance) as the final homophily.

### Ethical approval

Approval of all ethical and experimental procedures and protocols was granted by the Ethics Committee of Shandong Normal University.

## Results

### Descriptive statistics

**Figure 2:**
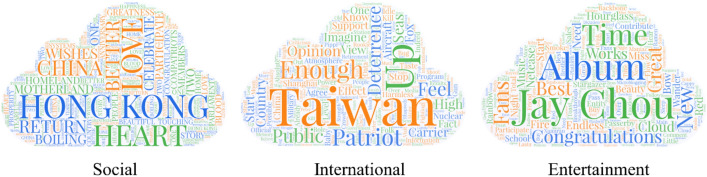
Keyword clouds for the three topics. We maintained the top 30 short videos with the largest number of comments under each topic as the set of events. Each topic corresponded to an interactive network formed by the commenting users of the 30 original short videos. The figure shows 3 topic keyword word clouds, and the size of the words is determined by the word frequency.

We used Python to crawl search results for three topics events on each platform. The raw information collected was filtered through data cleaning to remove about 25% of invalid information and information generated by paid internet trolls and bot accounts. The cleaned experimental data include 963 original short video informations, 391,676 comment texts, and 298,762 comment users and @user IDs. Then, the 963 original short videos were classified, and the top 30 videos with the highest number of comments under each topic were retained as the event set under each topic according to their number of comment users. Each topic corresponded to an interactive network formed by the commenting users of the 30 original short videos. The final experimental data consisted 90 original short videos from the three platforms, 82,426 comment texts and IDs of commenting users and @users. During the data-gathering procedure, we kept all target services’ terms of service, and the obtained dataset was anonymized. Keyword clouds for the three topics are shown in Fig. 2.

### Network structure and EC effect performance comparison

We used Python to compute the EC network consisting of nodes and edges for each platform. In terms of the number of ECs, Table 2 shows that the total number of ECs with two events under all topics across the three short video platforms (excluding the control group) is 3682, approximately four times the number of ECs with three short videos, and significantly fewer ECs with four and five events. Moreover, the number of members in ECs with two short videos is 34,564, which is roughly seven times that of ECs with three events. The number of users participating in ECs with four or five events is small and statistically insignificant. In order to prevent the data from being over-filtered, we finally selected two short videos of the EC as our research subjects.

**Table 2:** Number of ECs (ECs) formed by different short videos (SVs) under different platforms.

Platforms	Number of ECs formed by different number of SVs							
	Two SVs		Three SVs		Four SVs		Five SVs	
	ECs	Users	ECs	Users	ECs	Users	ECs	Users
Douyin	1303	17,892	382	1978	30	159	3	48
TikTok	1271	8049	233	1515	21	145	2	35
Blibili	1108	8623	315	940	15	93	1	11
Total	3682	34,564	930	4433	66	397	6	94

#### EC effect performance from platform dimension

**Figure 3:**
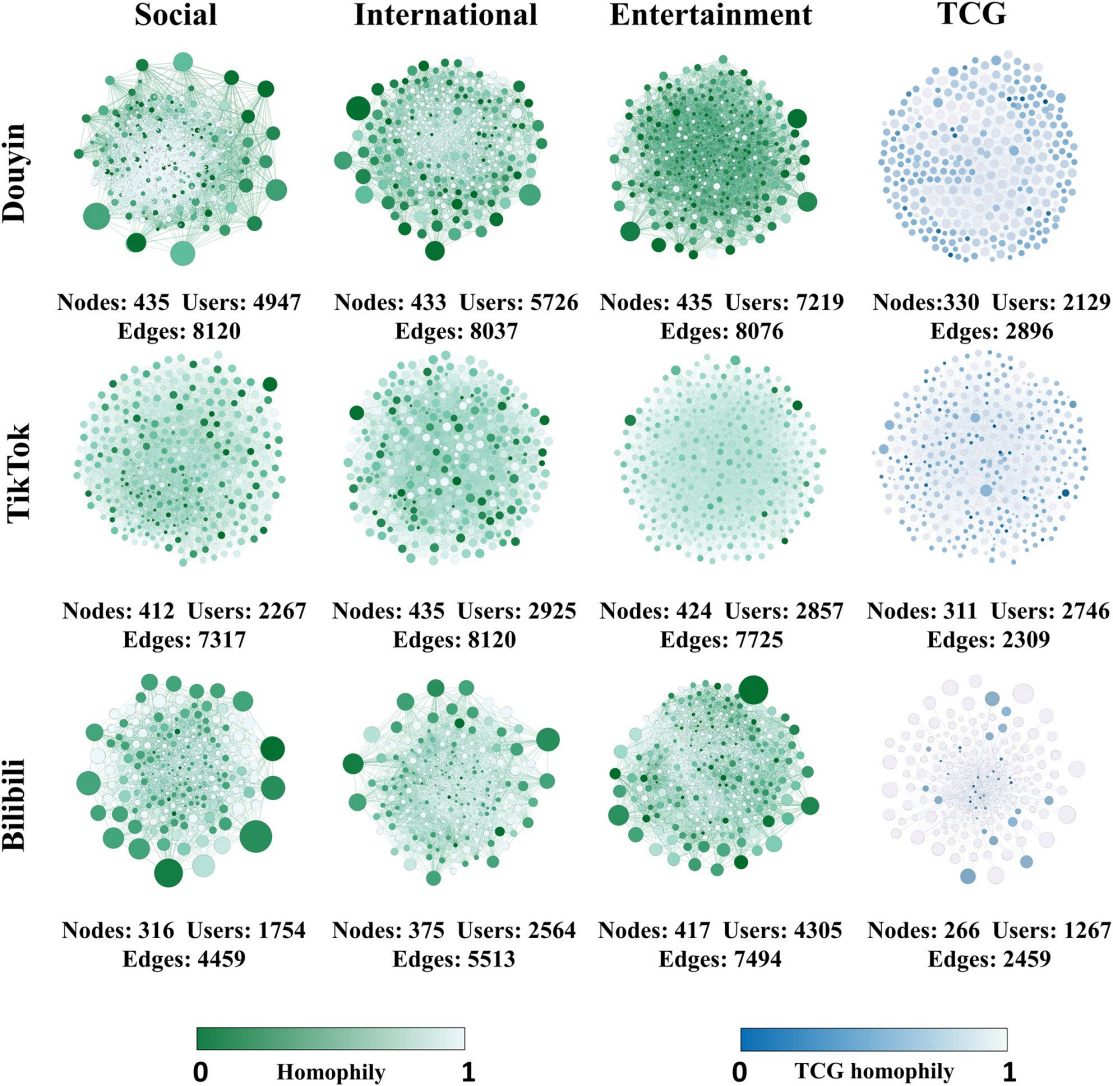
Visual structure diagrams for the ECs. Nodes represent ECs, and edges represent users involving two ECs. The size of the node represents the number of EC members and the color indicates the homogeneity level. When the homogeneity is close to 1/0, the color of the nodes is close to light/dark green. The blue network diagram symbolizes the control group, and the bluer the color represents the higher homogeneity, and vice versa.

We visualized the EC network using Gephi’s Force Atlas 2 layout algorithm. As shown in Fig. 3, a node represents an EC, and an edge represents a user involving two ECs. The node size represents the number of EC members, and the color indicates the homogeneity level. When the homophily is close to 1, the color of the node is close to light green, and when the homophily is close to 0, the color of the node is close to dark green. The blue network diagram is a control group.

*Size of ECs* Regarding the number of ECs, Douyin, TikTok and Bilibili are 1303, 1271 and 1108 respectively. All three platforms have significantly more ECs than the control group. In terms of the number of members participating in ECs, they are 17,892, 8049 and 8623, respectively. It is easy to see from the size of the nodes in Fig. 3 that TikTok is smaller and denser compared to the size of ECs on other platforms. The ECs on Douyin are significantly larger than those on other platforms in both number and size.

**Figure 4:**
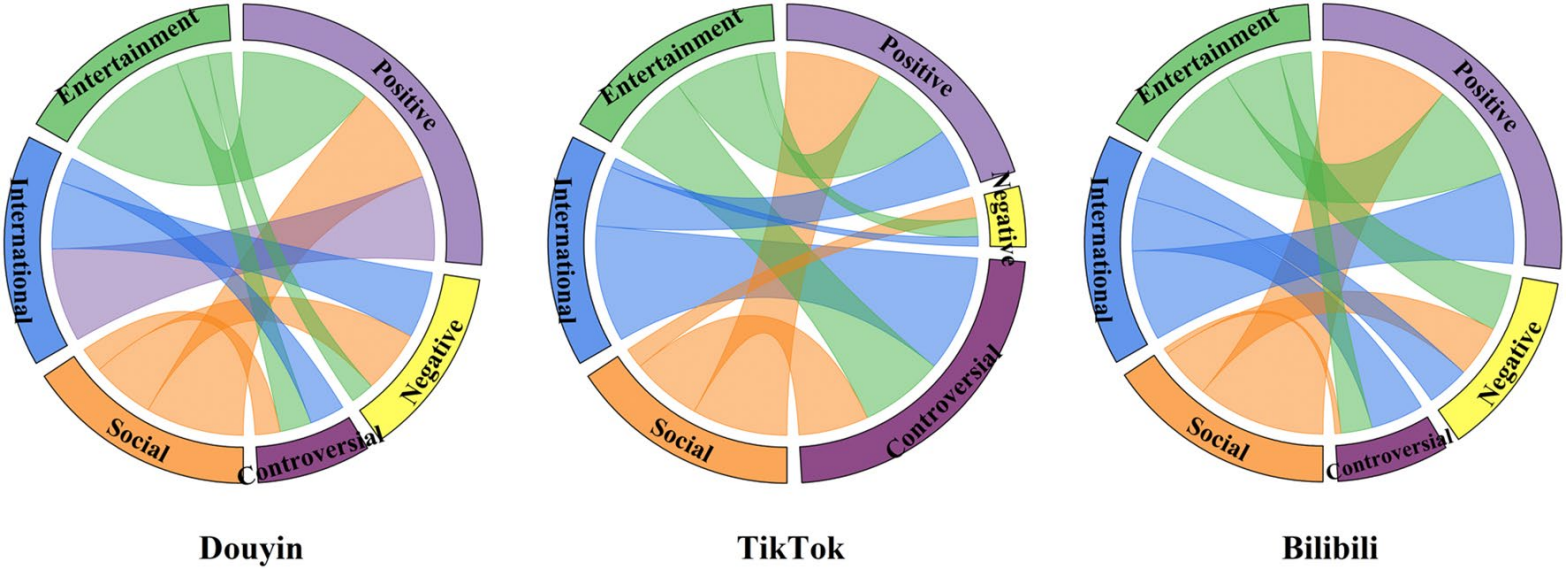
Chord diagram representation of platform dimension and topic dimension colored by sentiment. The three colors in the left semicircle indicate the three topics, and the three colors in the right semicircle indicate the set of positive, controversial, and negative EC types. The arc length indicates the percentage of ECs belonging to that type. The inner colored connecting bands indicate the flow and direction of the data relationships.

*Type of ECs* In Fig. 4, the three colors of the left semicircle indicate three different topics, and the different colors of the right semicircle indicate the set of three types of ECs: positive, controversial, and negative, respectively. The arc length indicates the total number of connections for all topics ECs belonging to that type of EC. The internal colored connection bands indicate the flow and order of magnitude of the data relationships. Douyin and Bilibili dominate with a positive EC ratio of about 50%. The proportions of controversial and negative ECs are about the same. In other words, these three platforms show a clear polarization from the dimension of EC types. However, TikTok has a larger proportion of controversial ECs, followed by positive ECs, and a very small proportion of negative ECs. TikTok has difficulty observing polarization in the dimension of EC type.

**Figure 5:**
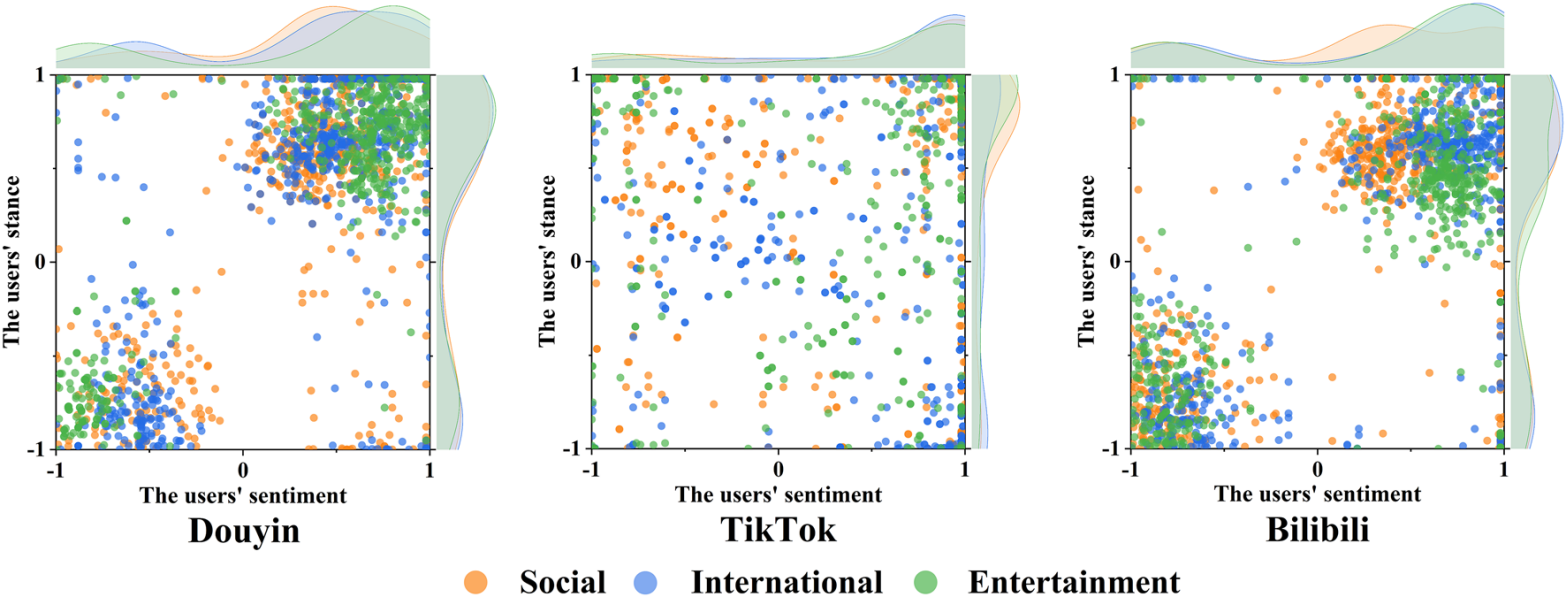
The distribution of users’ sentiment polarity in platform and topic dimensions. The X-axis indicates the user's sentiment score in the same EC, while the Y-axis indicates the user's stance score.

*Selective exposure* We further investigated how each EC displayed selective exposure across platforms. Figure 5 shows the sentiment polarity of the three platform EC members. The X-axis represents the sentiment polarity of users commenting on the first short video in the same EC, while the Y-axis indicates the sentiment polarity of the second one. We indicated three different topics in orange, blue and green, respectively. Not surprisingly, Douyin and Bilibili show a bimodal distribution trend. In contrast, TikTok shows only a single-peaked distribution of positive/agree sentiment/stance and a more discrete distribution compared to other platforms. This suggests that TikTok users are not divided into groups of opposite tendencies but form a general community.

**Figure 6:**
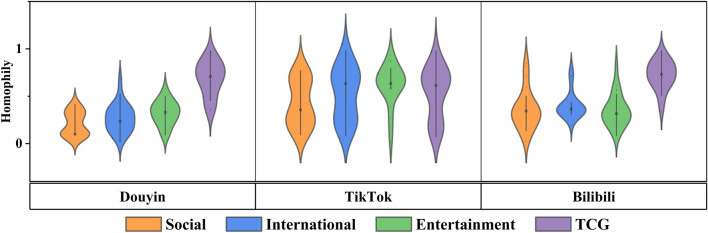
Homophily fiddle diagram representation of platform and topic dimensions. A wider fiddle chart indicates a higher percentage of users in that score range, and vice versa. The shape of the fiddle diagram demonstrates the trend of homogeneity distribution in the EC.

*Homophily* As shown in Fig. 3, it can be found from the EC node colors that the Douyin and Bilibili are closest to dark green. This reflects that the homogeneity of EC members on both platforms is closer to 0 (i.e., complete homogeneity, with only supporters or detractors present). TikTok shows a more homogeneous color distribution, which indicates that TikTok users do not exhibit significant homogeneity. And the homogeneity fiddle plot in Fig. 6 also shows the same distribution trend. These analyses indicate that short video propagation is skewed towards users with similar viewpoints on specific platforms (Douyin and Bilibili). In contrast, TikTok does not exhibit this effect in our investigation.

#### EC effect performance from topic dimension

Interestingly, from a topic perspective, the EC of each of the three short video platforms showcases different trends. This may be related to the differences in audience groups of different platforms.

*Size of ECs* Notice that, since the possible combinations of the number of ECs are $C_{30}^{2} = 435$. Therefore, the number of ECs > 400 per topic is a significant result. In Fig. 3, it is shown that in terms of the number of ECs, all short video platforms have significantly more ECs in all three topics than the control group. Douyin and TikTok have roughly the same distribution of the number of ECs in the three topics (> 400 per topic), but TikTok has fewer EC members. Bilibili, on the other hand, shows a lower number of ECs on the other two topics except entertainment. This is because Bilibili’s product positioning is pursuing a trendy cultural and entertainment community for more young people^30^. Our findings reflect to some extent the product positioning and user preferences of Bilibili. In terms of the number of EC members, Douyin has a slight advantage in the share of international topics. In the entertainment topic, all platforms show an absolute advantage in the number of EC members, which is in contrast to the social topic. To some extent, this reflects a preference in communication behavior among users of short video platforms. Users may focus more on the entertainment aspect of short videos, which is consistent with previous studies^7^.

*Type of ECs* In Fig. 4, the three different colored outer rings on the left represent three different topic sets of ECs, and the arc length represents the total number of connections of all EC types belonging to this topic. The three colors on the right represent three types of EC collections: positive, controversial, and negative. The inner colored connection bands indicate the flow direction and the size order between the different types of ECs and topics. Among the EC networks formed by the three platforms, positive ECs dominate in Douyin and Bilibili, followed by negative ECs, with a smaller proportion of controversial ECs, showing a significant polarization trend. TikTok, on the other hand, has a major share of controversial ECs on every topic, with a weak polarization trend.

*Selective exposure* In Fig. 5, there is a similar trend of selective exposure to different topics on the same platform. The EC members create two high-density clusters in the lower left and upper right regions across the three topics in Douyin and Bilibili. This suggests that consumers frequently consume content that reflects ideas similar to their own, demonstrating a blatantly selective exposure. On the other hand, TikTok’s distribution of user sentiment/stance polarity across the three topics is more discrete and lacks any discernible selective exposure. We discover that the platform dimension is more connected with the difference in selective exposure than the topic dimension.

*Homophily* Figure 6 shows the distribution of user homogeneity scores across different topics of ECs on the three platforms. Similar to selective exposure, we find a similar tendency of homogeneity distribution across topics on the same short video platform. As shown in Fig. 6, the average homogeneity scores of the three topics ECs of Douyin and Bilibili are below 0.4 (the closer the score is to 0, the more homogeneous it is). In contrast, the homogeneity distributions of TikTok and the control group show a wider spectrum. This result suggests that this result indicates that members of the same type of EC tend to hold similar views.

### The lifetime of short video EC

**Figure 7:**
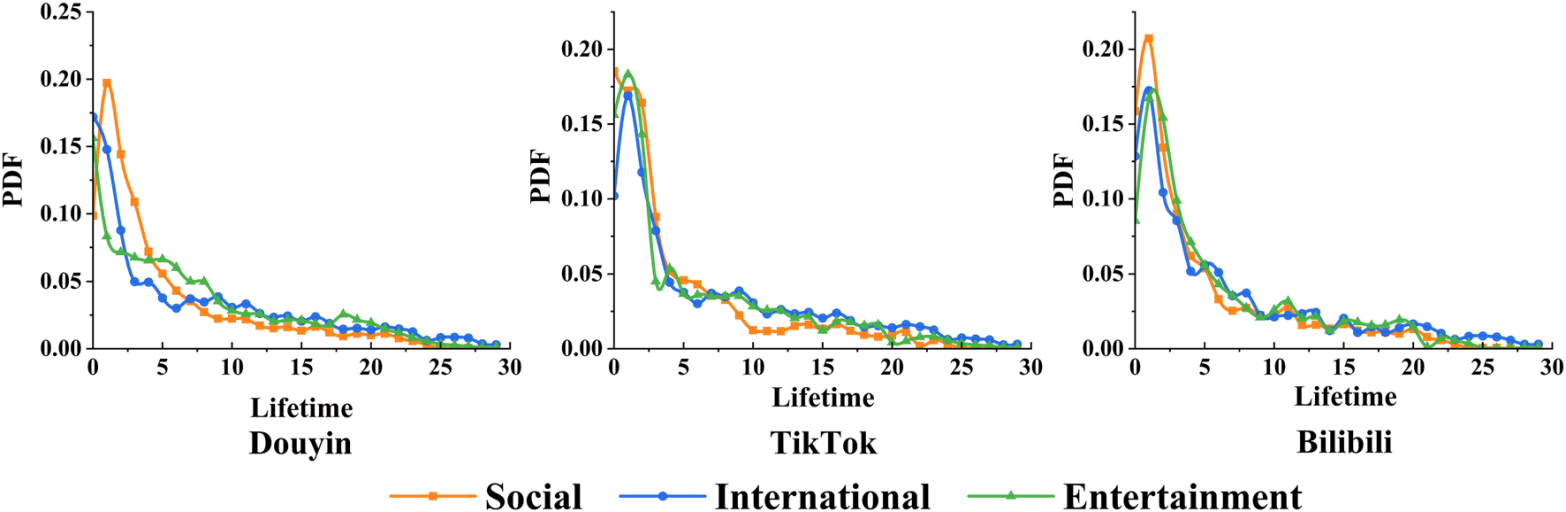
PDF of short video ECs’ lifetime. We calculated the lifetime as the length of time between the first user and the last user comment within the same short video. The probability density function (PDF) of the lifetime of a short video is in days as a time unit.

To examine the propagation patterns of short videos with different topics in ECs on different platforms, we measured the probability density function (PDF) of each lifetime of short video. We plotted the PDF of the lifetime (using days as time units) of short video propagation times with EC members. Figure 7 shows the probability density function (PDF) of the lifetime (using days as time units) of short videos. We calculated the lifetime as the length of time between the first user and the last user comment within the same short video. As shown in Fig. 7, the peak occurs in the first three days and the overall trend is a power-law distribution. This indicates that the pattern of information dissemination within the EC is similar over time, independent of differences in platforms and topics.

### The feature of short video EC member

In this section, we analyzed the features of short video EC members in four dimensions: user’s identity, spatial distribution, age, and comment time.

**Figure 8:**
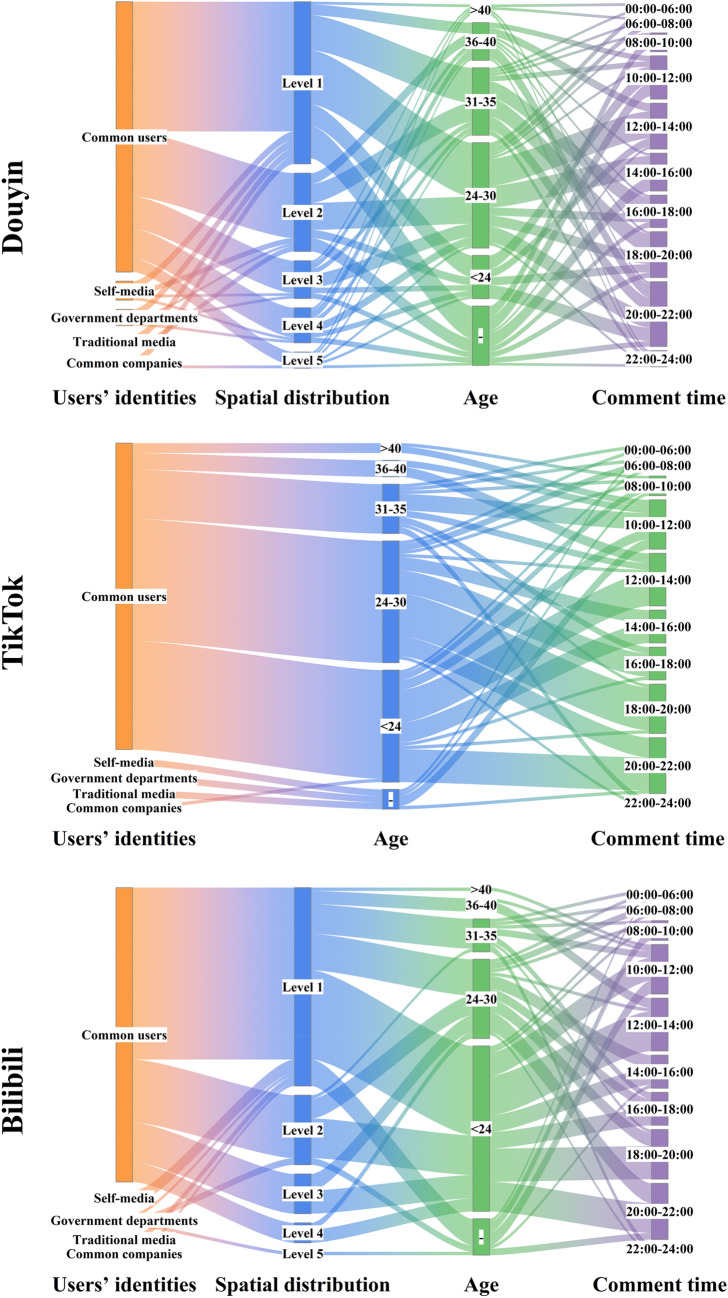
Sankey diagram representation of EC members’ identities, spatial distribution, age and comment time. The width of the lane is proportional to the degree to which it is weighted out and the inner colored connecting bands indicate the flow and direction of the data relationships.

In Fig. 8, the leftmost lane (orange) represents five different user’s identities: Common users, Self-media, Government departments, Traditional media, and Common companies. The width of the lane is proportional to its weighted out degree. Douyin’s users who participated in commenting accounted for 85.32%, 5.8%, 5.44%, 2.32%, and 1.12%, respectively. TikTok’s users accounted for 92.55%, 3.15%, 2.27%, 1.42%, and 0.61%, respectively. Bilibili's users accounted for 89.17%, 4.28%, 3.51%, 1.89%, and 1.15%, respectively. It is clear that ordinary users make up a major part of the EC members. It is worth mentioning that we did not identify self-media who participated in the comments as ordinary users (without the official certification logo of the platform). This situation is allowed in short video platforms, but this is very difficult to identify.

Next, we performed a spatial location analysis of the EC members marked with their true locations. Since TikTok geolocation access is non-public permission, we only test the variable of spatial location on Douyin and Bilibili. As shown in Table 3, we find by calculating the Kullback–Leibler Divergence between each two topics that there is a significant similarity between the spatial location of the EC members and a similar distribution to non-members (all KL Divergence values were less than 1). This indicates that there is no statistical correlation between ECs and users’ spatial location. Kullback–Leibler Divergence can be used to measure the similarity between two probability distributions; the closer the two probability distributions are, the smaller the KL divergence is^67,68^.

**Table 3:** User’s spatial location hierarchy criteria Kullback–Leibler divergence.

Topics		KL Divergence	
		Douyin	Bilibili
Social	International	0.3482	0.6412
International	Social	0.0390	0.9311
Social	Entertainment	0.0676	0.0782
Entertainment	Social	0.0317	0.0413
Social	TCG	0.5792	0.3981
TCG	Social	0.7146	0.2183
International	Entertainment	0.0317	0.1233
Entertainment	International	0.0213	0.2415
International	TCG	0.6491	0.0476
TCG	International	0.7269	0.0726
Entertainment	TCG	0.6246	0.0242
TCG	Entertainment	0.9978	0.0743

At the spatial scale of the whole China, the distribution of users of Douyin and Bilibili was similar, both relatively concentrated in developed eastern regions and surrounding provinces. From the proportion of user distribution in the figure, we can see obvious hierarchical differences in the concentration of short video EC members. The regions with denser user distribution are more consistent with the spatial pattern of national socio-economic development. As shown in Table 4, based on the total number of users in each region counted by both platforms as the ranking criteria, we divided the spatial locations into five levels on average (since there are 34 regions in total, the last level contains only 6 regions). Then, we can observe the relationship between spatial distribution and users’ identity, age, and comment time in Fig. 8.

**Table 4:** User’s spatial location hierarchy criteria.

Grade	Areas	Topic				Total (%)
		Social (%)	International (%)	Entertainment (%)	TCG (%)	
Level 1	Guangdong	12.07	17.46	15.22	2.70	47.45
	Henan	7.98	5.95	7.44	4.58	25.94
	Jiangsu	8.14	6.73	6.14	1.31	22.32
	Shandong	7.43	6.30	5.29	3.34	22.35
	Zhejiang	5.68	4.26	6.04	0.62	16.59
	Sichuan	5.48	4.87	5.44	13.19	28.98
	Anhui	4.29	5.13	4.12	3.38	16.91
Level 2	Hebei	4.29	4.34	4.39	2.50	15.52
	Guangxi	3.38	5.80	4.30	1.13	14.61
	Hubei	4.17	4.17	3.81	3.89	16.04
	Hunan	3.06	3.56	4.68	1.51	12.80
	Fujian	3.34	2.89	3.73	9.98	19.93
	Shaanxi	3.14	2.39	2.51	4.17	12.21
	Shanxi	2.90	2.45	2.18	3.06	10.59
Level 3	Liaoning	2.54	2.27	2.66	8.15	15.62
	Guizhou	2.50	2.54	2.29	2.18	9.51
	Jiangxi	2.18	2.27	2.70	0.99	8.15
	Beijing	2.70	2.07	1.83	2.54	9.14
	Yunnan	2.18	1.69	2.33	0.87	7.08
	Shanghai	1.95	2.10	1.73	0.62	6.39
	Chongqing	1.59	1.63	2.45	0.30	5.97
Level 4	Heilongjiang	1.51	1.19	1.52	5.43	9.65
	Xinjiang	1.55	1.22	1.01	1.40	5.19
	Jilin	0.99	1.63	1.48	3.14	7.25
	Inner Mongolia	0.87	1.57	1.34	3.09	6.88
	Tianjin	0.91	0.87	1.13	4.41	7.33
	Hainan	1.03	0.85	0.78	0.08	2.74
	Gansu	0.87	0.73	0.76	0.78	3.15
Level 5	Ningxia	0.52	0.47	0.37	1.55	2.90
	Qinghai	0.36	0.35	0.21	0.34	1.25
	Tibet	0.32	0.09	0.00	2.72	3.12
	Taiwan	0.04	0.12	0.10	5.98	6.24
	Macao	0.04	0.03	0.00	0.01	0.08
	Hong Kong	0.00	0.03	0.02	0.06	0.11

Meanwhile, we made statistics for the age of the EC members. As shown in the third lane (green) of Fig. 8, we divide the age groups of users according to the statistical age data: < 24, 24–30, 31–35, 36–40, and > 40. Douyin users accounted for: 18.76%, 28.97%, 25.23%, 17.74%, and 9.30%. TikTok users accounted for: 39.70%, 37.23%, 15.23%, 4.74%, and 3.10%. And Bilibili users accounted for: 61.6%, 22.88%, 10.13%, 3.56%, and 1.83%. This result indicates that there is a trend of younger members in the short video EC. In addition, as shown in the fourth lane (purple) of Fig. 8, the frequency of user interaction is highest from 10:00 to 14:00 at noon and from 18:00 to 22:00 at night. In addition, the frequency of user interaction is also relatively high during commuting time.

### The willingness to self-disclose of short video EC member

Self-disclosure refers to individuals voluntarily confiding their thoughts or showing their true behavior to the target person when interacting with others^69^. And it is proven to be prevalent in social media. In social networks, self-disclosure is a prerequisite for the formation of various human social relationships and is the basis for communication^70^.

In this section, we excluded EC members other than common users. And research on willingness to self-disclose was conducted only to the extent that platform privacy permitted. For each topic, we randomly selected 2000 EC members as the experimental group and 2000 non-members as the control group, respectively. According to the privacy setting rules of each platform, we divided the users’ willingness to self-disclose into four levels.

Douyin and TikTok:

- Level 1 was the absolute private account. Strangers need to request a follow or subscribe to the user before they can view the account content.
- Level 2 was a relatively private account. This type of user has opened the access rights of visitors to their personal videos, but not the access rights of “following”, “follower” and “like” of their personal homepage.
- Level 3 was relatively public account. These users can be subdivided into two types. The first is that the user has opened all permissions except “like” and the second type is that the user has opened the “like” permission, but not the “Follow” and “Fans” module access (because the visible functions of “following” and “follower” in the Douyin permission settings are bound).
- Level 4 was the absolute public account. This type of account opens access to all modules.

Bilibili:

Bilibili’s privacy setting rules are very different from the other two platforms. Therefore, we selected three privacy permissions related to the short video module as the metric: open my favorites, open recently liked videos and open my following list. If the user did not open all three permissions, it was judged as level 1; if one of the permissions was opened, it was judged as level 2, and the rest could be inferred by analogy.

**Figure 9:**
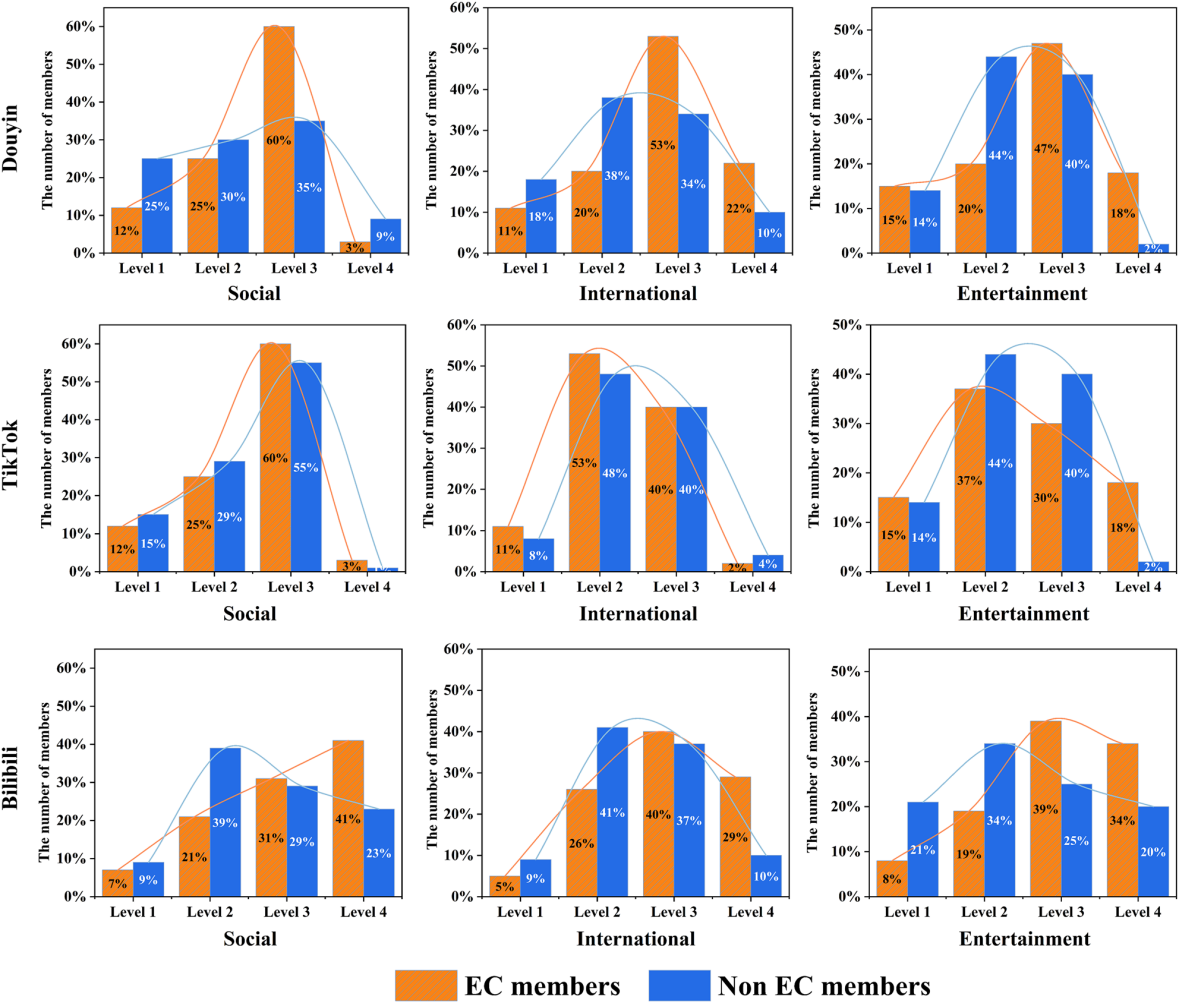
Willingness to self-disclose of users in the ECs and TCG. In the platform dimension, Douyin and Bilibili’s EC members’ willingness to self-disclose is mainly concentrated in Level 3 and Level 4, which is significantly higher than that of non-members. In contrast, in TikTok, where the EC effect was not obvious, we did not find significant differences between EC members and non-members.

Distributions of the willingness to self-disclose levels by EC members and non-members are shown in Fig. 9. In the platform dimension, Douyin and Bilibili’s EC members’ willingness to self-disclose is mainly concentrated in Level 3 and Level 4, which is significantly higher than that of non-members. In contrast, in TikTok, where the EC effect was not obvious, we did not find significant differences between EC members and non-members. Through the Mantel test, we find a statistically significant and highly positive correlation (*p* = 0.0082, *r* = 0.9756) between the correlation matrices of the EC members and non-members in TikTok. This implies that EC members are more willing to show themselves on the short video platform, which helps attract more users to follow them. In the topic dimension, we did not find any statistical correlation between the willingness to disclose privacy of EC members and non-members.

## Discussions

In this section, we will address our key findings as well as the academic and practical implications.

### Overall findings

In this study, we investigated the EC effect in short video distribution. To assess different user interaction behaviors, we conducted a comparative analysis of over 400,000 pieces of content from Douyin, TikTok and Bilibili on different topics (social, international and entertainment). The analysis focus on four main aspects: (1) the size of EC; (2) the type of EC; (3) selective exposure among EC members and (4) homophily in EC member interactions.

Our findings conclude that the EC effect maybe widely observed in some short video platforms. However, it is still related to the platform’s own functions and differences in positioning and audience groups. Both Douyin and Bilibili exhibit a strong EC effect (selective exposure and homophily), with the proportion of positive ECs dominating both platforms. In contrast, the EC effect on the TikTok platform is insignificant, and controversial ECs occupy more than half of the proportion. TikTok is a cross-lingual and cross-cultural short video platform that reaches 45 global countries and regions. In the analysis of selective exposure and homophily, we failed to find highly clustered groups, which is probably due to the language and cultural differences of users that limit the communication of like-minded groups, thus hindering the formation of ECs. On the other hand, we tried to investigate the impact of ECs on the spread of short videos. We calculated PDFs of ECs’ lifetimes, where the lifetime is here computed as the temporal distance (in days) between the first and second comment of a user in each EC. Despite the varying platforms and topics, we find that the temporal sharing patterns in each category are similar. We also tried to analyze users’ identity, spatial location, age, and comment time, but none of them were found to be significantly different from non-members. To our surprise, we found that EC members are more willing to self-disclose than non-members. They tend to actively present themselves to attract their peers to join the discussion.

### Theoretical implications

The theoretical contributions are as follows: first, as mobile internet technology has grown, consumers now have access to a new era of short videos as well as new social media features owing to a variety of short video platforms^19^. Previous studies on the EC effect have focused more on traditional social media platforms, and few studies have explored the form and extent of the EC effect during information dissemination on short video platforms. This study makes a bold attempt in this area.

Second, we compared the similarities and differences of the EC effect in the interaction behavior of users of different short video platforms and topics, broadening the study of the impact of social features of short video platforms on user behavior. Meanwhile, this study extracts positive, negative, and controversial ECs to scrutinize their contents and derive meaningful values to convey varied details on pertinent concerns.

Third, our research reveals that linguistic and cultural barriers may prevent like-minded groups from communicating on short video platforms, making it impossible for users to create polarised clusters and limiting the formation of ECs.

Fourth, this study enriches the literature related to ECs by exploring the impact of the EC effect on the spread of short videos in several dimensions: lifetime of EC, feature of EC member, and users’ willingness to self-disclose.

### Practical implications

The study results can provide strategic guidance for managers of short video platforms to utilize the EC effect for content optimization, expand audience reach, and improve user satisfaction, leading to stakeholder communication and cooperation.

First, the EC effect can be used to create a new monitoring system that analyses web content to learn more about the feelings and concerns of the general population. This research can be applied generally to examine data from various short video platforms including Kwai, Likee, and Vimeo.

Second, high-traffic short videos have a directing influence on viewers’ psyche and conduct. It will have an extremely harmful effect on society if short video makers solely desire to achieve commercial advantages through their short videos and overlook social benefits. Therefore, the EC effect of short video platforms can be used to target anomalous groups quickly (e.g., ECs with a high proportion of negative sentiment and large size) for positive interventions in the early stages of short video distribution.

Third, as a new type of social media, short video platforms inevitably have some fake news or misinformation. In order to control the adverse effects brought by such information, the EC effect can be used to apply different intervention strategies to users with different sentiments. At the same time, platform managers can appropriately use the preferences of different topic EC members for personalized information recommendation services, aiming to avoid information vacuums and prevent cognitive deficiencies and narrow biases.

## Conclusion

The short video platforms are distinguished by content-based interactions that heavily rely on uniquely personalised videos selected by the feed algorithm of the platforms. In socio-technical systems, algorithms are typically imperceptible mechanisms that have the power to change how we interact with one another and even push users into a polarized EC. This study systematically explored the EC effect in disseminating short videos on different topics (social, international, and entertainment) on three platforms: Douyin, TikTok and Bilibili. The study confirmed a significant EC effect in the commenting behavior of Douyin and Bilibili, but TikTok did not perform significantly. By comparing the performance of the echo chamber effect on the different platforms and topics, we conclude that the pattern of information dissemination within the EC is similar and independent of platforms and topics. Moreover, EC members commonly display themselves in order to catch the attention of their peers and that cultural differences can hinder the growth of ECs.

Although the above results were obtained in this study, there are still some limitations. First, the data collection period is limited to 30 days to control the effect of time variables. In future studies, we will consider the relationship between the dissemination of information and ECs for longer periods. Second, the main users of Douyin and Bilibili are concentrated in Asia, and the selected topic events also attract more attention and discussion from Asian users. Therefore, we cannot risk making generalizations about ECs on short video platforms. Third, unfortunately, since some short video platforms do not publicly release information on video retweets and favorite users, we limit our current study to the dimension of comment networks. Previous studies have found that retweet networks have a stronger EC effect than comment networks. As data permissions allow, future work will include extensively exploring the impacts of user interaction behaviors on EC effects, for example, subscription behavior of users, across a broader range of short video platforms and topic events. Finally, the data used in this study are in multiple national languages (predominantly Chinese), and future studies will try to explore the laws of the existence of EC effect in multimodal language in order to draw general generalized conclusions, which will be very meaningful research.

## Data Availability

The datasets used and/or analysed during the current study available from the corresponding author on reasonable request.
